# A Large-Scale Structural Classification of Antimicrobial Peptides

**DOI:** 10.1155/2015/475062

**Published:** 2015-04-27

**Authors:** Hao-Ting Lee, Chen-Che Lee, Je-Ruei Yang, Jim Z. C. Lai, Kuan Y. Chang

**Affiliations:** Department of Computer Science and Engineering, National Taiwan Ocean University, Keelung 202, Taiwan

## Abstract

Antimicrobial peptides (AMPs) are potent drug candidates against microbial organisms such as bacteria, fungi, parasites, and viruses. AMPs have abundant sequences and structures, two fundamental resources for bioinformatics researches, but analyses on how they associate with each other are either nonexistent or limited to partial classification and data. We thus present A Database of Anti-Microbial peptides (ADAM), which contains 7,007 unique sequences and 759 structures, to systematically establish comprehensive associations between AMP sequences and structures through structural folds and to provide an easy access to view their relationships. 30 distinct AMP structural fold clusters with more than one structure are detected and about a thousand AMPs are associated with at least one structural fold cluster. According to ADAM, AMP structural folds are limited—AMPs only cover about 3% of the overall protein fold space.

## 1. Introduction

Antimicrobial peptides (AMPs) are potent drug candidates against microbial organisms such as bacteria, fungi, parasites, and viruses. Up to date, more than 10 AMPs have entered clinical trials [1]. Due to the importance, several databases dedicated to AMPs were released in the past few years. Some databases are species-specific such as BACTIBASE [2], BAGEL2 [3], DADP [4], PenBase [5], and PhytAMP [6]; some curate a broad spectrum of species such as AMPer [7], APD2 [8], CAMP2 [9], DAMPD [10], Defensins Knowledgebase [11], and YADAMP [12]. The sizes of these databases range from hundreds to a couple of thousand AMP sequences. However, none of these databases contains all.

Understanding sequence-structure relationships is important for AMP-based drug design. However, one of the major limitations in AMP databases is poorly utilizing structural information. Like AMP sequences, various AMP structures have been resolved. Classified by secondary structures, four traditional AMP structures are alpha helices, beta strands, loop structures, and extended structures [13, 14]. An alternative structural classification using peptide backbone torsion angles also shows many different AMP folds [1]. Few AMP databases such as APD2 have attempted to associate AMP sequences with their secondary structures. However, none has established associations between AMP sequences and AMP structural folds. Examining AMP tertiary structures would help us understand AMPs better and enhance potential antimicrobial drug discovery.

In this work, we present A Database of Anti-Microbial peptides (ADAM) (available at http://bioinformatics.cs.ntou.edu.tw/ADAM). ADAM collects AMPs comprehensively and establishes associations systematically between AMP sequences and structures. Integrated from various sources, ADAM contains the most complete AMP sequences and structures. ADAM not only allows biomedical researchers to search basic AMP information but also provides an easy access to link AMP sequences to structures and vice versa.

## 2. Data Collection and Methods

### 2.1. AMP Sequences

**Figure 1:**
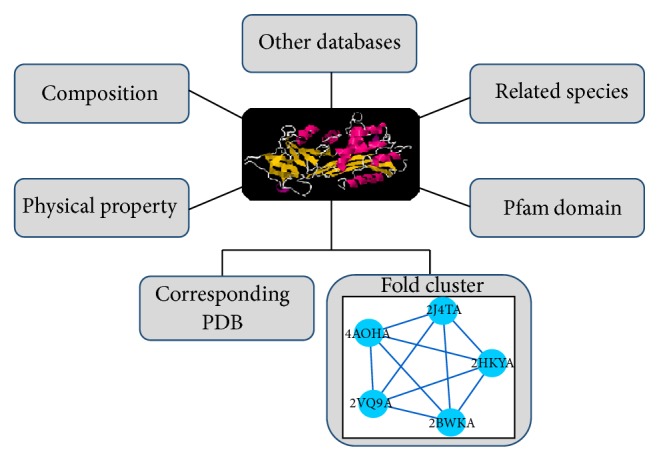
Simplified conceputal diagram of ADAM.

ADAM contains 7,007 unique AMP sequences extracted from twelve databases (Figure 1). The twelve databases include APD2 [8], AVPpred [15], BACTIBASE [2], BAGEL3 [3], CAMP2 [9], DADP [4], DAMPD [10], HIPdb [16], PenBase [5], PhytAMP [6], RAPD [17], and YADAMP [12]. The AMP sequences in ADAM were mostly derived from natural sources, covering a broad spectrum of species such as archaea, bacteria, plants, and animals. 2497 out of the 7,007 sequences have been validated experimentally and recorded in literature.  Table 1 compares the AMPs of the twelve databases. The CAMP2 contains the most overlapping sequences among the large AMP databases such as APD2, CAMP2, DAMPD, DADP, and YADAMP. For species-specific AMP databases, AVP and HIPdb are found to contain less overlapping sequences.

Each unique AMP sequence was assigned an ADAM ID. The ADAM ID is linked to the basic AMP information, structural view, physicochemical properties, amino acid composition, and external resources. The structural view displays the best corresponding PDB structure and, if available, the representative PDB structure of the fold cluster which this AMP sequence belongs to. The physicochemical properties list peptide length, net charge, instability index^∗∗^, aliphatic index^∗∗^, and grand average of hydropathicity index^∗∗^. The composition is the ratio of each amino acid in the AMP. The other resources are linked to PDB, CATH, SCOP, Pfam, and other AMP databases associated with this AMP (^∗∗^see Supplementary Material available online at http://dx.doi.org/10.1155/2015/475062).

### 2.2. AMP Structures

The AMP structures were obtained by running BLAST of the experimentally validated AMP sequences against the Protein Data Bank [18]. 408 sequences had 759 matching structures with either 100% sequence identity or at least 90% identity sequence with the *E*-value < 10^−5^. Each matching structure was annotated by SCOP v1.75B [19] and CATH v4.0 [20]. Because not every AMP structure had CATH or SCOP annotation, one could not determine all unique AMP structural folds simply based on these annotations.

Tables 2 and 3 record the number of the AMP structures according to CATH v4.0 and SCOP v1.75B, respectively. Four hierarchical levels of CATH are class, architecture, topology, and homologous superfamily; four levels of SCOP are class, fold, superfamily, and family. The topology level of CATH corresponds to the fold level of SCOP. The AMP structures appear at the entire four fundamental CATH classes (Table 2) and seven SCOP classes (Table 3). Within 759 AMP structures, 40 out of 1375 CATH folds (Table 2) and 47 out of 1390 SCOP folds (Table 3) are found. These AMP structures cover about 3% of the protein fold space defined by CATH and SCOP.

### 2.3. AMP Structural Fold Clusters

**Figure 2:**
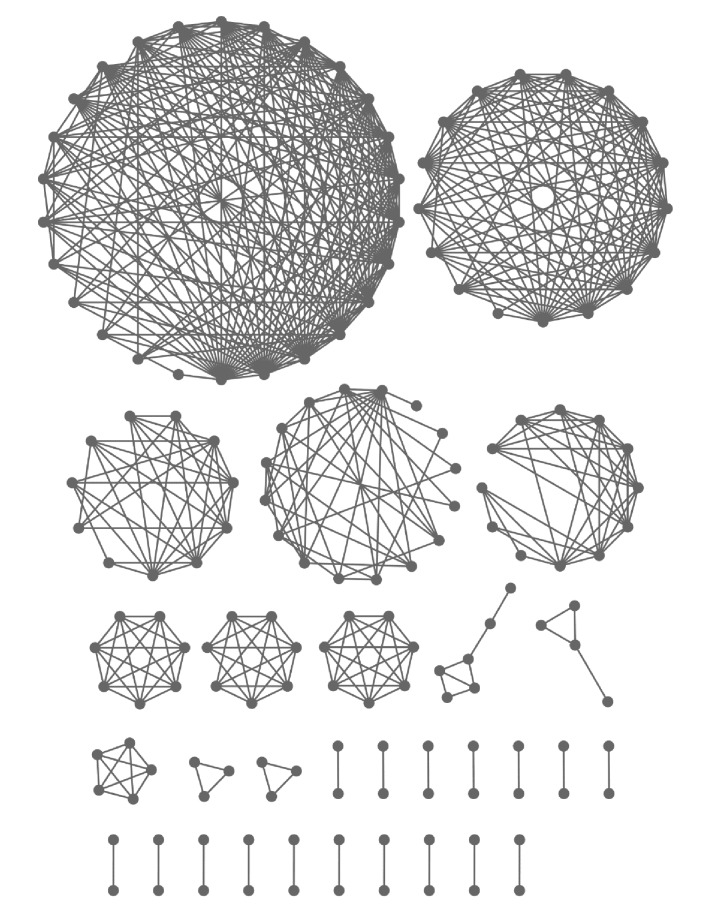
Nework representation of AMP structral fold clusters.

A graph-based clustering procedure was applied for accessing the unique AMP folds. In this graph, the vertices represent AMP structures and there is an edge between two vertices if the two AMP structures are similar. The AMP structures came from the previous BLAST results. Only 264 best matching structures were collected under more stringent selection conditions. Each AMP is allowed to have at most one best matching structure, and multiple AMPs can map to the same AMP structure. The similarity of two AMP structures was then measured by TM-score, whose value ranges from 0 to 1 [21]. An edge exits if its TM-score > 0.5, which indicates that the two structures should belong to the same fold [22]. 136 AMP fold clusters were formed with 30 clusters containing more than one AMP structure, as shown in Figure 2. The top 10 common AMP structural folds with CATH and SCOP annotations are listed in Table 4. The structural fold clusters can have the same CATH and SCOP annotations as cluster #1 in Table 4. One CATH fold can map to multiple SCOP folds as cluster #4 in Table 4; one SCOP fold can also map to multiple CATH folds as cluster #9 in Table 4. Note that some AMP structures have neither CATH nor SCOP annotation.

The vertices represent the AMP structures and an edge between two vertices exists if the TM-score > 0.5, indicating the two structures as the two verctices fall into the same fold [22]. Among the 136 fold clusters in ADAM, 30 of them which contain more than one structure are displayed here.

### 2.4. AMP Structures Associated with ADAM Sequences

From AMP sequences to structures, AMP structures were obtained by performing BLAST on the experimentally validated AMPs against PDB. From AMP structures to ADAM sequences, about one-eighth of the ADAM sequences, over a thousand sequences, were found to associate with the AMP structures, which were determined by running BLAST against the best matching AMP structures with the *E*-value < 10^−5^. Here we list the top 10 common Pfam domains and families [23] found in the experimentally validated AMPs and their associations with the AMP structural fold clusters (Table 5). Out of these common Pfam domains and families, seven of them fall within the top 10 AMP structural folds. Table 5 also indicates that no structures are available for the AMPs with Pfam family antimicrobial_1.

## 3. Implementations and Results

ADAM was built using AppServ 2.6.0. The Apache HTTP server was applied, the server-side scripts were written in PHP, and the database was built by MySQL.

### 3.1. Multiple Search Capabilities

ADAM offers multiple search capabilities, which can be classified into two basic categories: sequence search and structural search. Each AMP entry is assigned an ADAM ID, which would have a unique sequence and, if found, a corresponding structure. The sequence search covers the direct information of an AMP sequence, including the description, source species, sequence length, and Pfam domain. ADAM which focuses on AMP structure and sequence information does not contain all of the information that other AMP databases provide. Therefore, external links to other AMP databases are also provided in ADAM. In addition, the structural search allows users to retrieve the AMP information associated with specific PDB structures or ADAM fold clusters.

### 3.2. Structure-Sequence Cluster Browsing

ADAM offers 136 AMP fold clusters built by TM-score for browsing. Each structure in the AMP cluster is annotated by CATH, SCOP, and Pfam, if available. The AMP structures from all of the clusters occupy about 3% of the protein fold space defined by CATH and SCOP. Each cluster would list the associated AMP sequences.

For example, ADAM cluster #1 (AC_001) is a cluster of 26 structures associated with 207 AMP sequences. Detailed information can be found at Table S1. These structures in this cluster gathered by TM-score are consistently classified into the same CATH fold, alpha-beta 2-layer sandwich defensin A-like structure, and the same SCOP fold, small protein knottins. SCOP further classifies these structures into four different SCOP families. In addition, this AMP structural fold is found to associate with six different Pfam domains, including antimicrobial_6, defensin_2, gamma-thionin, toxin_2, toxin_3, and toxin_37, which supports that different sequences which fold into the same structure could behave similarly. Another interesting example is ADAM cluster #5 (AC_005), which contains 53 AMP sequences involved with cyclotide Pfam family. Within this cluster, only four structures are annotated by SCOP. All of the four structures are again classified into the same SCOP fold, knottins, but fall into multiple SCOP families.

ADAM also allows users to extract the relevant AMP structures according to CATH or SCOP classification by the underneath hyperlinks. In fact, both structure-to-sequence and sequence-to-structure browsing can be performed in ADAM.

Each AMP cluster is further examined. An interesting phenomenon is observed that peptides in one AMP cluster consistently belong to the same mechanism of microbial killing, either transmembrane pore formation or metabolic inhibition of intracellular targets [24], suggesting that AMP structures may play a role in the killing action. For example, the AMPs in ADAM cluster #3 (AC_003) belong to the mechanism of transmembrane pore formation; those in ADAM cluster #6 (AC_006) are the metabolic inhibitors for the intracellular targets.

## 4. Discussions

ADAM, which is a comprehensive AMP database, provides an easy access to AMP sequences, structures, and their relations. Two distinct characters of ADAM are its size and sequence-structure analysis. ADAM contains 7,007 unique AMP sequences and 759 structures. To our knowledge, this is the first comprehensive study to analyze various AMP structural folds. Our analysis demonstrates that AMP structures cover about ~3% of the overall CATH or SCOP folds. Biologically this infers more than one scheme for AMPs to fight microbes. The results also indicate that AMP structural folds are limited. The majority of the protein structural folds lack antimicrobial activities.

The development of ADAM raises some interesting research topics, which are beyond the scope of this study, still waiting to be explored. To name a few, for example, Table 5 shows that little is known of the structure of Pfam family antimicrobial_1. Such AMP structures need to be resolved by X-ray crystallography or NMR spectroscopy; Table 4 demonstrates a prolonged discussion that CATH and SCOP classifications are not always consistent with each other [21]. The best approach to annotate protein structure is still to be determined. Despite sequence differences between Pfam antimicrobial_2 and DD_K domains, the two domains somehow share the same alpha-helical structural fold: how the two different domains maintain the same structural fold as well as antimicrobial activities still needs more studies.

ADAM, which offers complete AMP sequence and structure information, can benefit a number of different AMP researches such as biomimetics in drug development, comparative immunomics, and structure-function analysis. For example, ADAM cluster #1 (AC_001) has 26 structures associated with 207 AMP sequences (Table S1). Not every structure in the cluster has annotations, but those which do belong to the same CATH and SCOP fold, matching with six different kinds of Pfam families. Such information can help to identify key elements for antimicrobial drug design.

## Supplementary Material

The content of the Supplementary Material can be classified into three main categories: (1) an example of ADAM fold cluster, (2) the technical descriptions for aliphatic index^**^, instability index^**^, and hydropathicity^**^, and (3) the frequently asked questions about ADAM. In more detail, Table S1 lists ADAM fold cluster AC_001 with 26 AMP structures associated with 207 unique AMP sequences. Figure S1 and S2 illustrate how to browse through ADAM either from AMP sequence-structure or AMP structure-sequence. Figure S3 and S4 show the basis of the AMP prediction tools using support vector machines and hidden Markov models, which are provided in ADAM.

## Figures and Tables

**Table 1:** Comparison of overlapping identical sequence counts of the twelve AMP databases.

	APD	CAMP	DADP	DAMPD	YAD	BACTIB	BAGEL	PenBase	PhytAMP	RAPD	AVP	HIPdb
APD	**2436**	2100	744	376	1601	86	39	1	107	55	56	33
CAMP	2100	**3052**	858	586	1994	122	56	1	145	71	65	33
DADP	744	858	**1792**	220	772	0	0	0	0	5	13	11
DAMPD	376	586	220	**1068**	528	31	70	5	19	10	18	11
YADAMP	1601	1994	772	528	**2782**	113	43	1	60	67	76	49
BACTIBASE	86	122	0	31	113	**204**	52	0	0	10	0	1
BAGEL	39	56	0	70	43	52	**431**	0	0	0	1	0
PenBase	1	1	0	5	1	0	0	**28**	0	0	0	0
PhytAMP	107	145	0	19	60	0	0	0	**272**	3	10	0
RAPD	55	71	5	10	67	10	0	0	3	**119**	9	5
AVP	56	65	13	18	76	0	1	0	10	9	**604**	156
HIPdb	33	33	11	11	49	1	0	0	0	5	156	**744**

**Table 2:** Structural classification of the AMPs according to CATH v4.0 classification.

	Class	Architecture	Topology	Homologous superfamily
ADAM	4	11	40	41
CATH 4.0	4	40	1375	2738

**Table 3:** Structural classification of the AMPs according to SCOP v1.75B.

	Class	Fold	Superfamily	Family
ADAM	7	47	53	72
SCOP 1.75B	11	1390	2220	4609

**Table 4:** Top 10 common AMP structural folds annotated by CATH and SCOP.

AMP structural folds	CATH			SCOP	
Fold cluster ID	Class	Architecture	Topology	Class	Fold
1	Alpha beta	2-layer sandwich	Defensin A-like	Small proteins	Knottins

2	Mainly beta	Beta barrel	OB fold	Alpha and beta proteins (a + b)	IL8-like

3	Mainly alpha	Up-down bundle	Single alpha-helices involved in coiled-coils or other helix-helix interfaces	Peptides	Antimicrobial helix

3	—	—	—	Peptides	Liposaccharide-binding protein CAP18

3	—	—	—	Peptides	Peptide hormones

4	Alpha beta	Roll	Antimicrobial peptide, beta-defensin 2; chain A	Small proteins	Defensin-like

5	—	—	—	Small proteins	Knottins

6	Mainly alpha	Orthogonal bundle	Histone, subunit A	All alpha proteins	Histone-fold

7	Mainly alpha	Orthogonal bundle	Lysozyme	Alpha and beta proteins (a + b)	Lysozyme-like

8	Alpha beta	2-layer sandwich	Crambin	Small proteins	Crambin-like

9	Mainly alpha	Orthogonal bundle	NK-lysin	All alpha proteins	Saposin-like

9	Mainly alpha	Up-down bundle	Bacteriocin As-48; chain A	All alpha proteins	Saposin-like

10	Alpha beta	Roll	P-30 protein	Alpha and beta proteins (a + b)	RNase A-like

**Table 5:** Top 10 common Pfam domains and families associated with the AMP structural folds.

	Pfam	AMP structural fold cluster ID
1	Antimicrobial_2	3
2	Antimicrobial_1	NA
3	Defensin_beta	4
4	Gamma-thionin	1
5	Cyclotide	5
6	Defensin_2	1
7	Defensin_1	4
8	Bacteriocin_II	33
9	Cecropin	106
10	DD_K	3
