# Supplementary material for: A Large-Scale Structural Classification of Antimicrobial Peptides
Source: Biomed Res Int. 2015 Apr 27;2015:475062. doi: 10.1155/2015/475062 (PMC4426897; doi:10.1155/2015/475062)
Supplement: Supplementary file 1 — The content of the Supplementary Material can be classified into three main categories: (1) an example of ADAM fold cluster, (2) the technical descriptions for aliphatic index**, instability index**, and hydropathicity**, and (3) the frequently asked questions about ADAM. In more detail, Table S1 lists ADAM fold cluster AC_001 with 26 AMP structures associated with 207 unique AMP sequences. Figure S1 and S2 illustrate how to browse through ADAM either from AMP sequence-structure or AMP structure-sequence. Figure S3 and S4 show the basis of the AMP prediction tools using support vector machines and hidden Markov models, which are provided in ADAM. [file 475062.f1.pdf]

SUPPLEMENTARY INFORMATION

A large-scale structural classification of antimicrobial peptides

Hao-Ting Lee, Chen-Che Lee, Je-Ruei Yang, Jim Z. C. Lai, Kuan Y. Chang*

ADAM is at http://bioinformatics.cs.ntou.edu.tw/ADAM.

Tables

Table S1. ADAM's Cluster AC_001 with 26 AMP structures associated with 207 unique AMP sequences.

Figures

Figure S1. Basic AMP information provided by ADAM.
Figure S2. AMP structure-sequence relationships displayed in ADAM's browsing page.
Figure S3. Average amino acid composition of the AMPs in ADAM.
Figure S4. Pfam coverage of the twelve AMP databases.

**Note:

The following descriptions of the terms are direct quotos from our previous study [1].

Aliphatic Index:

The aliphatic index, the relative volume of aliphatic residues in a peptide, is calculated as follows:

$$\mathrm{AI} = \mathrm{X}_{\mathrm{Ala}} + a * \mathrm{X}_{\mathrm{Val}} + b * (\mathrm{X}_{\mathrm{Leu}} + \mathrm{X}_{\mathrm{Ile}})$$

where *a* and *b* are the constants, which represent the relative volume of valine and leucine or isoleucine to alanine. X_{Ala}, X_{Val}, X_{Leu}, and X_{Ile} are the fractions of alanine, valine, leucine and isoleucine multiplied by 100, respectively [2].

Instability Index:

The instability index, an estimate of peptide stability, is calculated as follows:

$$\mathrm{II} = \frac{10}{L}\sum_{\mathrm{i}=1}^{\mathrm{i}=\mathrm{L}-1} DIWV(\mathrm{X}_{\mathrm{i}}, \mathrm{X}_{\mathrm{i}+1})$$

where *L* is the length of peptide and *DIWV* from the study by Guruprasad *et al.* is an instability weight value of a dipeptide starting at position i [3]. Peptides with II values greater than 40 are considered to be unstable.

Hydropathicity:

Grand average of hydropathicity index (GRAVY) is used to represent the hydrophobicity value of a peptide, which calculates the sum of the hydropathy values of all the amino acids divided by the sequence length. GRAVY was calculated using the hydropathy values from Kyte and Doolittle [4]. Positive GRAVY values indicate hydrophobic; negative values mean hydrophilic.

Table S1. ADAM's Cluster AC_001 with 26 AMP structures associated with 207 unique AMP sequences.

PDB				CATH			SCOP			
ID	Chain	#Seq	Pfam	Class	Architecture	Topology	Class	Fold	Superfamily	Family
1AYJ	A	126	Gamma-thionin	Alpha Beta	2-Layer Sandwich	Defensin A-like	Small proteins	Knottins (small inhibitors, toxins, lectins)	Scorpion toxin-like	Plant defensins
1BK8	A	2	Gamma-thionin	Alpha Beta	2-Layer Sandwich	Defensin A-like	Small proteins	Knottins (small inhibitors, toxins, lectins)	Scorpion toxin-like	Plant defensins
1FJN	A	4	Defensin_2	NA	NA	NA	Small proteins	Knottins (small inhibitors, toxins, lectins)	Scorpion toxin-like	Defensin MGD-1
1GPT	A	2	Gamma-thionin	Alpha Beta	2-Layer Sandwich	Defensin A-like	Small proteins	Knottins (small inhibitors, toxins, lectins)	Scorpion toxin-like	Plant defensins
1I2U	A	11	Toxin_3	Alpha Beta	2-Layer Sandwich	Defensin A-like	Small proteins	Knottins (small inhibitors, toxins, lectins)	Scorpion toxin-like	Insect defensins
1ICA	A	11	Defensin_2	Alpha Beta	2-Layer Sandwich	Defensin A-like	Small proteins	Knottins (small inhibitors, toxins, lectins)	Scorpion toxin-like	Insect defensins
1JKZ	A	1	Gamma-thionin	Alpha Beta	2-Layer Sandwich	Defensin A-like	Small proteins	Knottins (small inhibitors, toxins, lectins)	Scorpion toxin-like	Plant defensins
1L4V	A	12	Defensin_2	Alpha Beta	2-Layer Sandwich	Defensin A-like	Small proteins	Knottins (small inhibitors, toxins, lectins)	Scorpion toxin-like	Insect defensins
1MM0	A	1	Toxin_37	NA	NA	NA	Small proteins	Knottins (small inhibitors, toxins, lectins)	Scorpion toxin-like	Insect defensins
1MR4	A	5	Gamma-thionin	Alpha Beta	2-Layer Sandwich	Defensin A-like	Small proteins	Knottins (small inhibitors, toxins, lectins)	Scorpion toxin-like	Plant defensins
1MYN	A	3	Gamma-thionin	Alpha Beta	2-Layer Sandwich	Defensin A-like	Small proteins	Knottins (small inhibitors, toxins, lectins)	Scorpion toxin-like	Insect defensins
1N4N	A	2	Gamma-thionin	Alpha Beta	2-Layer Sandwich	Defensin A-like	Small proteins	Knottins (small inhibitors, toxins, lectins)	Scorpion toxin-like	Plant defensins

1OZZ	A	18	Toxin_3	Alpha Beta	2-Layer Sandwich	Defensin A-like	Small proteins	Knottins (small inhibitors, toxins, lectins)	Scorpion toxin-like	Insect defensins
1P00	A	9	NA	Alpha Beta	2-Layer Sandwich	Defensin A-like	Small proteins	Knottins (small inhibitors, toxins, lectins)	Scorpion toxin-like	Insect defensins
1P0A	A	13	Toxin_3	Alpha Beta	2-Layer Sandwich	Defensin A-like	Small proteins	Knottins (small inhibitors, toxins, lectins)	Scorpion toxin-like	Insect defensins
1TI5	A	3	Gamma-thionin	Alpha Beta	2-Layer Sandwich	Defensin A-like	NA	NA	NA	NA
1ZFU	A	2	Defensin_2	NA	NA	NA	NA	NA	NA	NA
2A9H	E	5	Toxin_2	NA	NA	NA	Small proteins	Knottins (small inhibitors, toxins, lectins)	Scorpion toxin-like	Short-chain scorpion toxins
2B68	A	1	Defensin_2	NA	NA	NA	NA	NA	NA	NA
2E2F	A	3	Antimicrobial_6	NA	NA	NA	NA	NA	NA	NA
2GL1	A	2	Gamma-thionin	Alpha Beta	2-Layer Sandwich	Defensin A-like	NA	NA	NA	NA
2KGQ	A	3	NA	Alpha Beta	2-Layer Sandwich	Defensin A-like	NA	NA	NA	NA
2KSK	A	2	Gamma-thionin	NA	NA	NA	NA	NA	NA	NA
2LJ7	A	2	Gamma-thionin	Alpha Beta	2-Layer Sandwich	Defensin A-like	NA	NA	NA	NA
2LR5	A	1	Defensin_2	NA	NA	NA	NA	NA	NA	NA
2LT8	A	1	Defensin_2	NA	NA	NA	NA	NA	NA	NA

ADAM's Cluster AC_001 consists of 26 structures and 207 sequences. Among these 26 structures, six structures have neither CATH nor SCOP annotation. These with CATH or SCOP consistently fall into the same fold. Note: The homologous family field of CATH is not displayed, for all of them are empty here.

Frequently Asked Questions:

Q: How does ADAM provide AMP sequence-structure or structure-sequence relationships?
A: The goal of ADAM is to provide an easy access to link AMP sequences to structures and vice versa on a comprehensive AMP dataset. We assume that one AMP sequence can be mapped to at most one AMP structure and one AMP structural fold can consist of many structures.

To examine AMP sequence-structure relationship, ADAM offers users to search information directly based on AMP sequences. The other search options include ADAM ID, name or keyword, sequence length, taxonomy, links to other AMP databases, and Pfam family as well as PDB ID and ADAM cluster ID. A sample result page is as shown in Supplementary Fig. S1. Through this information page, users can find out what kind of structure the AMP sequence has and which structural fold it belongs to.

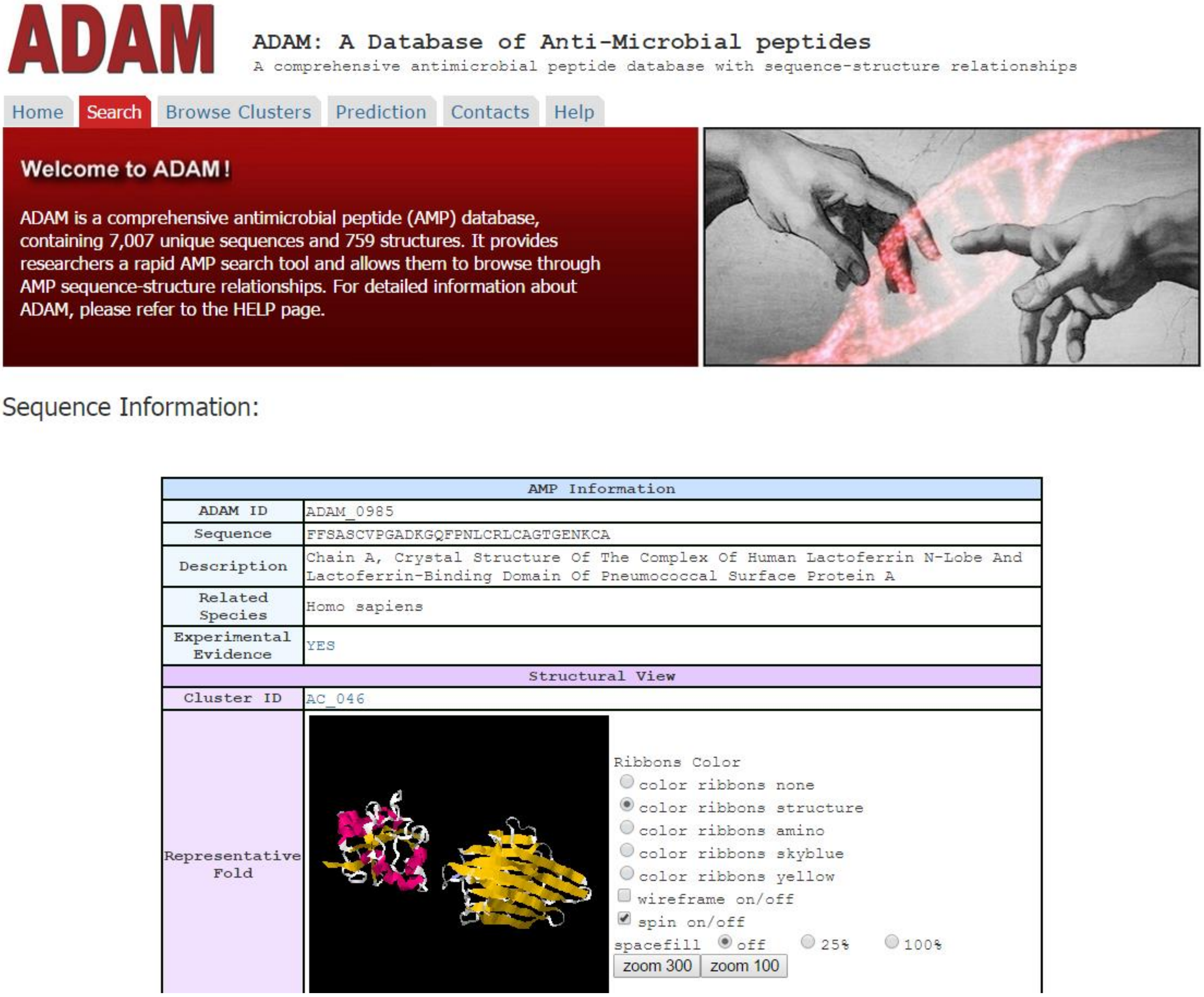

Figure S1. Basic AMP information provided by ADAM. Here is a partial page of the basic information. Links on this page allow users to explore the relationship from AMP sequence to structure.

ADAM provides three structural-fold approaches to examine AMP structure-sequence relationships: (1) AMP fold clusters by TM-score, (2) CATH, and (3) SCOP. The default method is AMP fold clusters because not every PDB structure is annotated by CATH or SCOP and the similarities of any two structures can be

measured by TM-score. As described previously, a graph-based clustering approach using TM-score is utilized to build the AMP fold clusters [5]. In this graph, the vertices represent the AMP structures and an edge between two vertices exists if the two AMP structures are similar, which is determined by TM-score. Thus the structures within the same cluster share the same structural fold, indicated by TM-score. ADAM lists all the associated AMP sequences within the same fold directly from the browsing page under the detail button (Supplementary Fig. S2). Besides, ADAM allows users to browse the relationships through CATH or SCOP. The hyperlinks underneath the CATH or SCOP annotation in the browsing page would display the AMP structures under the particular classification at any of the four levels of CATH (class, architecture, topology, and homologous superfamily) or SCOP (class, fold, superfamily, and family). All the three approaches can view an AMP structure or an AMP structural fold with the associated AMP sequences.

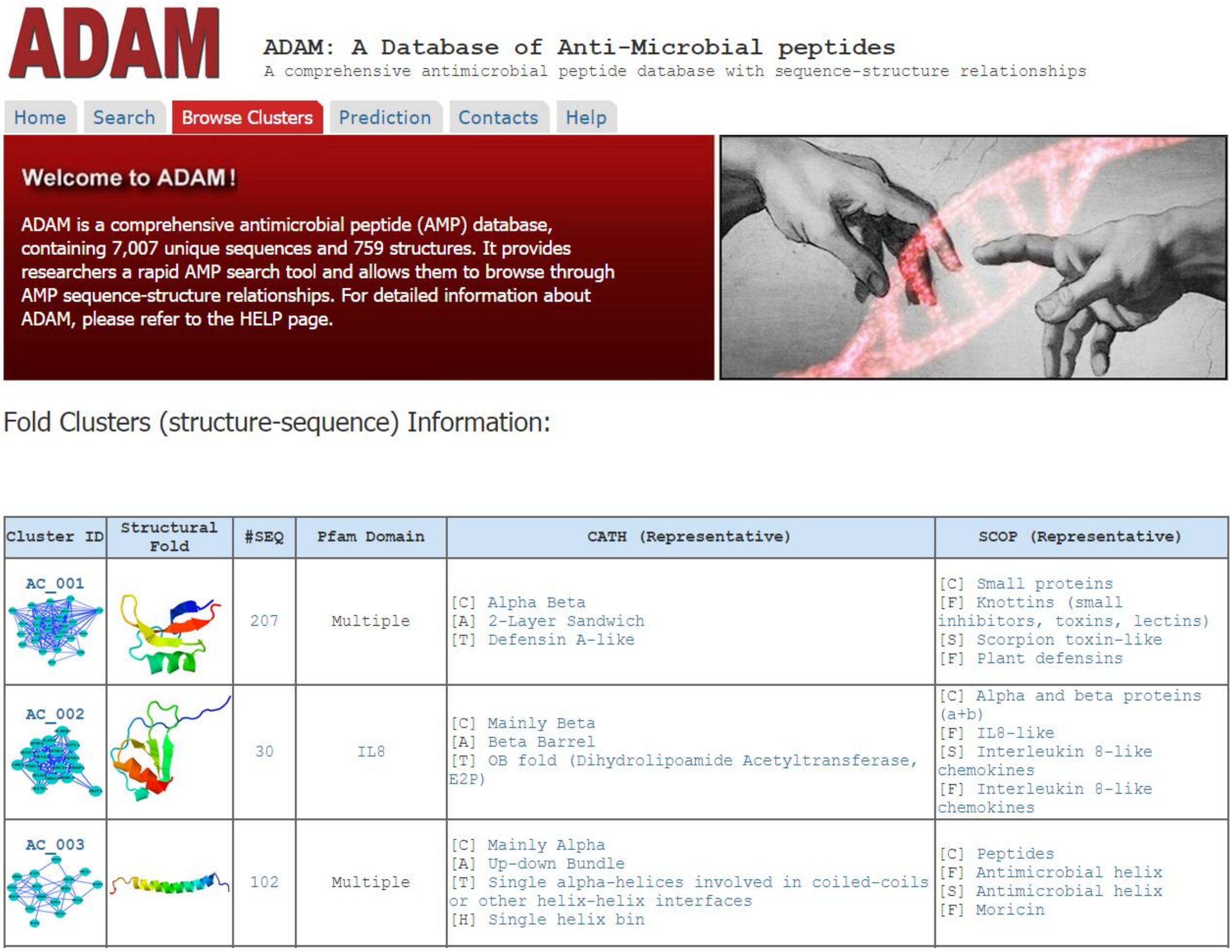

Figure S2. AMP structure-sequence relationships displayed in ADAM's browsing page. Three approaches to view the relationships in ADAM are AMP fold clusters (Cluster ID), CATH, and SCOP. The hypertexts of Cluster ID, CATH, and SCOP would list the PDB structures, the number of the AMP sequences, Pfam domains, and fold annotation. Besides, the hypertext of the number of the sequences would list all the associated AMP sequences.

Q: Why did ADAM apply AMP fold clusters to annotate the AMP structures in addition to CATH and SCOP?

A: The main reason why ADAM applied AMP fold clusters by TM-score is that ADAM needs an effective way to classify all of the AMP structures. There are several options to conduct this analysis. CATH and SCOP are our first choice. However, not every PDB structure is annotated by CATH or SCOP. Over a third of these structures have neither CATH nor SCOP annotation. TM-score provides a relatively rapid and reliable method to measure the similarity of any two PDB structures.

Q: ADAM focuses on AMP structural folds, but can ADAM display AMP structure-sequence relationship through AMP secondary structure?

A: Yes, ADAM can. By default, ADAM displays the relationships through fold cluster, CATH, and SCOP. In fact, the first level of CATH (class) and SCOP (class) refers to the secondary structure. The hyperlinks are available to visualize the comprehensive AMP relationship by secondary structure.

Supplementary information concerning AMP Prediction tools:

Built on the AMP sequences of ADAM, two prediction tools are available on ADAM (http://bioinformatics.cs.ntou.edu.tw/adam/tool.html) to identify potential AMP sequences. Support vector machines (SVM) and hidden Markov models (HMM) are used separately to build these tools. The SVM predictor is based on AMP composition and the HMM predictor utilizes the Pfam domains.

SVM

Our SVM model was trained on the AMP sequences of ADAM, using amino acid composition as the learning features (Supplementary Fig. S3). LIBSVM (http://www.csie.ntu.edu.tw/~cjlin/libsvm/) [6] was chosen to build our SVM prediction model. A radial basis function kernel was applied to the SVM model, whose optimal cost and gamma parameters for the kernel were determined by LIBSVM.

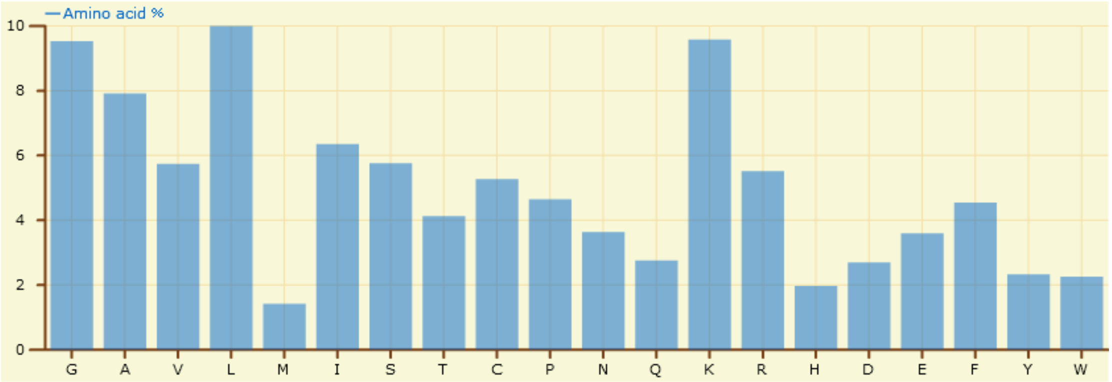

Figure S3. Average amino acid composition of the AMPs in ADAM.

HMM

Our HMM predictor was built on two kinds of HMM profiles: (1). The Pfam domains found in the AMP sequences of ADAM. (2). Additional HMM profiles for the AMP sequences without any Pfam domains in ADAM. Here a conserved domain analysis was performed on the twelve AMP databases using the Pfam 27.0 database [7]. It is known that the Pfam domains cover nearly 80% of all the proteins, but the Pfam coverage among the twelve AMP databases was only around 40% ~ 70% (Supplementary Fig. S4). HIPdb

has the least Pfam coverage, only around 17.2%. Relative low coverage indicates that AMPs are still waiting to be explored. In our analysis, 236 Pfam families are found in the ADAM sequences. For the ADAM sequences without any Pfam families, 30 repeating patterns were collected to form the additional HMM profiles. Our HMM predictor utilizes the two kinds of HMM profiles to identify potential AMPs though sequence homolog.

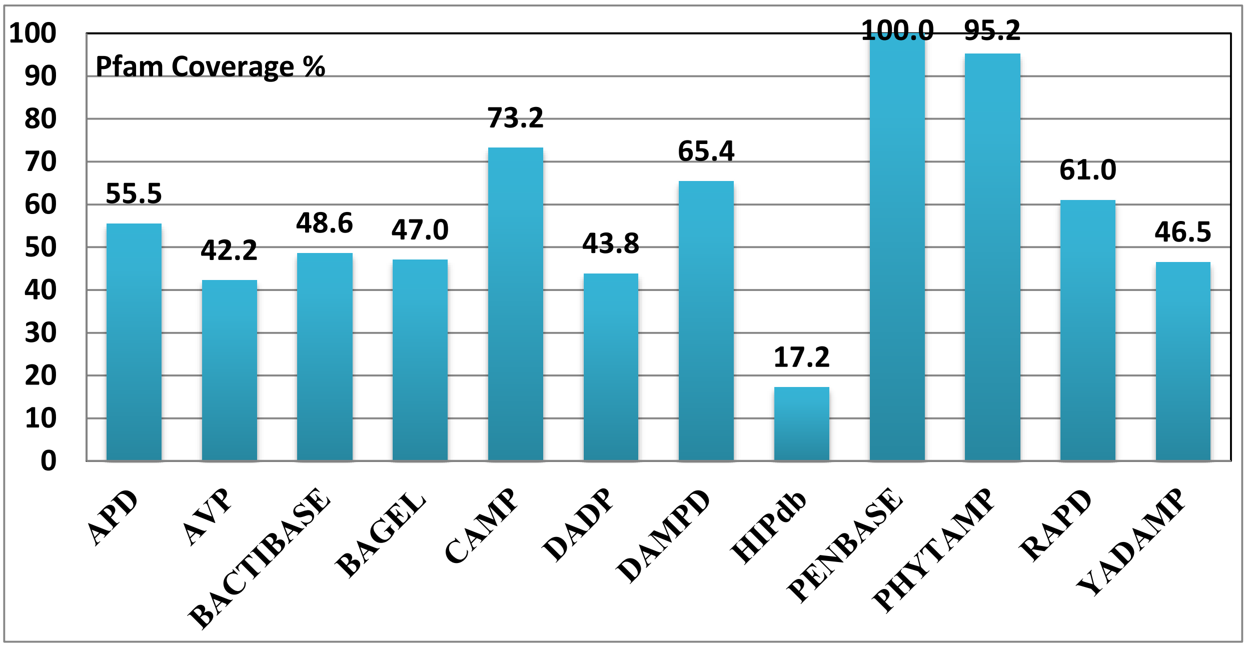

Figure S4. Pfam coverage of the twelve AMP databases.

REFERENCES

[1] K. Y. Chang and J. R. Yang, "Analysis and prediction of highly effective antiviral peptides based on random forests," *PLoS One,* vol. 8, p. e70166, 2013.

[2] A. Ikai, "Thermostability and aliphatic index of globular proteins," *J Biochem,* vol. 88, pp. 1895-8, Dec 1980.

[3] K. Guruprasad, B. V. Reddy, and M. W. Pandit, "Correlation between stability of a protein and its dipeptide composition: a novel approach for predicting in vivo stability of a protein from its primary sequence," *Protein Eng,* vol. 4, pp. 155-61, Dec 1990.

[4] J. Kyte and R. F. Doolittle, "A simple method for displaying the hydropathic character of a protein," *J Mol Biol,* vol. 157, pp. 105-32, May 5 1982.

[5] Y. Zhang and J. Skolnick, "Scoring function for automated assessment of protein structure template quality," *Proteins,* vol. 57, pp. 702-10, Dec 1 2004.

[6] C.-C. Chang and C.-J. Lin, "LIBSVM: A Library for support vector machines," *ACM Transactions on Intelligent Systems and Technology,* vol. 2, 2011.

[7] M. Punta, P. C. Coggill, R. Y. Eberhardt, J. Mistry, J. Tate, C. Boursnell*, et al.*, "The Pfam protein families database," *Nucleic Acids Res,* vol. 40, pp. D290-301, Jan 2012.
